# A Case of Miliary Perinatal Tuberculosis in a Preterm Newborn Infant Presenting as Peritonitis

**DOI:** 10.7759/cureus.8036

**Published:** 2020-05-09

**Authors:** Artsiom Klimko, Alienor Brandt, Maria-Iulia Brustan

**Affiliations:** 1 Division of Physiology and Neuroscience, Carol Davila University of Medicine and Pharmacy, Bucharest, ROU; 2 Pediatrics, Carol Davila University of Medicine and Pharmacy, Bucharest, ROU; 3 Pediatrics Section, Marie Curie Children's Emergency Hospital, Bucharest, ROU

**Keywords:** congenital tuberculosis, peritonitis, splenic tuberculosis

## Abstract

Perinatal tuberculosis (TB) is a rare disease with nonspecific clinical symptoms that can result in delayed treatment, and contribute to high morbidity and mortality. We report a case of perinatal TB in a 25-day-old newborn who presented with fever, respiratory distress, disseminated intravascular coagulation (DIC), and marked abdominal distension with hepatosplenomegaly. Further workup revealed culture negative sepsis, peritonitis, and diffuse nodular infiltrate in the lungs, liver, and spleen. After an extensive diagnostic workup for potential gastrointestinal causes of sepsis, the diagnosis of TB was finally established via paracentesis and maternal testing. Our objective is to draw attention to the multifaceted clinical manifestations of perinatal TB despite classically being associated with pulmonary symptoms; extensive gastrointestinal involvement should not exclude perinatal TB from the differential during the workup of culture-negative sepsis.

## Introduction

Tuberculosis (TB) is one of the leading causes of morbidity and mortality worldwide and is the most common cause of death from an infectious disease [1]. According to estimates from the World Health Organization (WHO), the incidence of new TB cases began to decline in 2015-2016 by an estimated 1.9% per year, but globally, there are still approximately four million new cases reported each year [1]. Romania is not endemic for TB, but has the highest TB incidence in the European Union, despite considerable progress since 2002 [2]. Perinatal TB is notoriously difficult to diagnose due to its wide array of nonspecific symptoms, with clinical suspicion for this disease rising only after patients deteriorate despite broad-spectrum antibiotic therapy [3]. Due to immune system immaturity, TB in the pediatric population tends to quickly progress to more severe disease forms, such as meningitis and military TB; if treatment and diagnosis is delayed, mortality approaches 50% [4].

## Case presentation

A 25-day-old newborn male was brought to the ED with complaints for mild fever (38.7°C), refusal to feed, and abdominal distention. He was born prematurely at 33 weeks via spontaneous vaginal delivery to a previously healthy primigravid 24-year-old woman and cried immediately after birth. He weighed 2100 g and Apgar scores were 7 and 8 at 1 and 5 min, respectively. At birth he received the anti-hepatitis B vaccine and Bacillus Calmette-Guérin (BCG) vaccine on day of life 20. He was then discharged. Upon admission to the ED, the newborn appeared irritable, hypotonic, and in obvious respiratory distress. Systemic examination revealed an enlarged abdomen that was tympanic to percussion, as well as hepatosplenomegaly; the liver and spleen were palpable three and two centimeters below the rib cage, respectively. Other findings are summarized in Table 1 and compared against the 10 most common signs and symptoms seen in children diagnosed with perinatal TB after 14 days of age [5].

 

**Table 1:** The 10 most common clinical manifestations of congenital TB in children diagnosed after 14 days of age compared to findings in our case. TB, tuberculosis

Clinical manifestation	Frequency (%)	Our case findings
Fever	84	Yes
Hepatomegaly	69	Yes
Cough	65	Yes
Respiratory distress	57	Yes
Cyanosis	43	No
Moist pulmonary rales	39	No
Abdominal distension	22	Yes
Pallor	20	Yes
Triple restriction	20	No
Poor appetite	16	Yes

**Figure 1:**
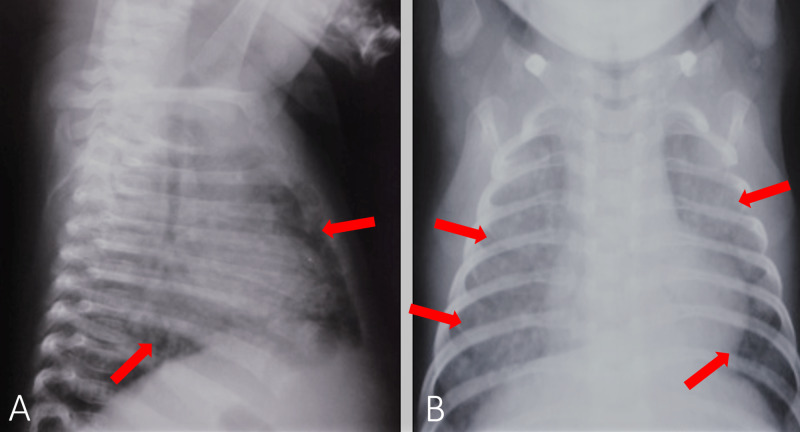
Chest radiograph. (A) Lateral view - diffuse opacifications of lung fields. (B) Anterior-posterior view - bilateral diffuse nodular infiltrate.

**Figure 2:**
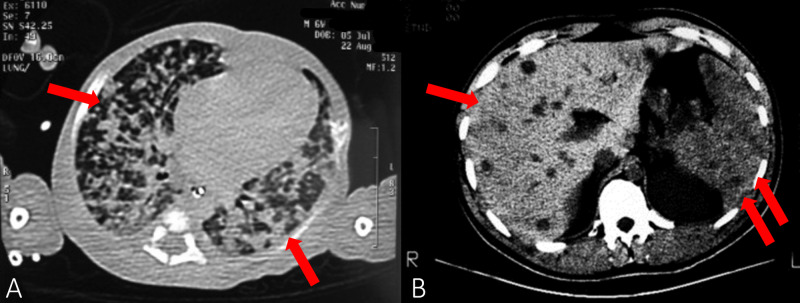
Axial CT. (A) Chest CT showing bilateral opacities. (B) Abdominal CT showing multiple nodular hepatic lesions (arrow) and enlarged spleen (double arrows).

Lab tests revealed anemia (hemoglobin 6.8 g/dL), leukopenia (WBC count 2.6 × 109/L), thrombocytopenia (32,000 platelets/μL), metabolic acidosis, elevated acute phase reactants (C-reactive protein 107 mg/L and procalcitonin >2 ng/mL), and elevated prothrombin and partial thromboplastin time. Blood cultures, urinalysis, stool samples, and cerebrospinal fluid (CSF) analyses were unrevealing, as were the serology tests for toxoplasma, rubella, cytomegalovirus, and herpes simplex virus infections. Gastric aspirate was bilious, but was otherwise unremarkable. It is important to note that at this time, polymerase chain reaction (PCR) analysis of the gastric aspirate for TB was not made due to low clinical suspicion. A chest radiograph showed diffuse reticulonodular infiltration of both lung fields with pneumonia-like changes (Figure 1), while the CT showed numerous nodules throughout the lung fields (Figure 2A). Abdominal ultrasonography revealed hepatomegaly with multiple diffuse micronodular lesions with hypogenic contours, which was later further evaluated with CT (Figure 2B); abdominal ultrasound also revealed hilar hepatic lymphadenopathy with a maximum diameter of 26/15 mm. The spleen was enlarged, numerous lesions were noted similar to the hepatic ones, and some free liquid in the peritoneal cavity with some debris was also present. Transfontanellar ultrasonography did not reveal any abnormalities.

Based on the clinical presentation, a presumptive diagnosis of late-onset culture-negative neonatal sepsis was made and empiric treatment with ampicillin, vancomycin, meropenem, with antifungals was initiated. In addition, the DIC was treated with intravenous immunoglobulin, fresh frozen plasma, packed red blood cell, and platelet transfusions. The hepatosplenomegaly, distended abdomen, ascites, biliary stasis, and hepatic microabscesses raised suspicion of a gastrointestinal cause of sepsis and further workup was initiated. The pathologies that were suspected included necrotizing enterocolitis, spontaneous intestinal perforation, neonatal appendicitis, and other anatomical or functional causes of intestinal obstruction and subsequent enterocolitis (e.g., congenital aganglionic megacolon, intestinal neuronal dysplasia, volvulus, meconium ileus, intussusception, etc.).

Abdominal radiography revealed marked distention of the entire abdomen, but pneumatosis intestinalis, air-fluid levels, pneumoperitoneum, and portal venous gas (pneumatosis hepatis) were all absent, ruling out necrotizing enterocolitis. Barium enema showed complete homogenous filling of the rectum and colon without the Neuhauser sign or transition zones that would suggest meconium ileus or congenital aganglionic megacolon. A negative workup for gastrointestinal causes of sepsis, in addition to the continued clinical and paraclinical deterioration of the patient placed TB as the most likely cause for the disseminated miliary granulomas. A chest radiography of the mother was done immediately and yielded findings suggestive for pulmonary TB, prompting a consult from a pneumology institute. She was screened for TB; a tuberculin skin test (TST) was positive induration >15 mm and the chest radiograph showed a small pleural effusion with an opacity in the right upper lung field. The mother was isolated from the infant and started on a nine-month isoniazid regimen, as per treatment guidelines for latent TB infection (LTBI). To their knowledge, the parents were not aware of any sick contacts; TST was negative for other family members, including the father. In the meantime, paracentesis was performed on the newborn, yielding a turbid-appearing sample with a white blood cell count of 320/mm3, serum-ascites albumin gradient (SAAG) < 1.1 mg/dL, and positivity for all three modified Light’s criteria for exudative peritoneal effusions to confirm tuberculous peritonitis [6]. The neonate’s clinical condition improved after he was started on anti-TB therapy (rifampin, isoniazid, and pyrazinamide).

## Discussion

The most recent criteria for establishing the diagnosis of congenital TB were put forth by Cantwell et al. and they include a proven tuberculous lesion in a newborn, in addition to at least one of the following: (i) lesions in the first week of life; (ii) a primary focus of caseating hepatic granulomas; (iii) maternal genital tract or placental TB; and (iv) exclusion of postnatal transmission through sick contacts [4]. Our patient possibly qualifies for the diagnosis of congenital TB, as there were proven tuberculous lesions with hepatic granulomas, especially as transmission via the umbilic vein or aspiration of infected amniotic fluid could be a fitting explanation for the extensive gastrointestinal involvement. However, due to lack of placental samples and postnatal isolation of the neonate from the mother who had a LTBI, it is difficult to support that diagnosis.

Manifestation of TB in the perinatal period was always surrounded by controversy, as there are inherent limitations in ascertaining whether the infection was acquired in a congenital or postpartum manner -- due to this reason, perinatal TB is now the preferred term [3]. Additionally, Singh et al. cited that perinatal TB can be either of congenital or neonatal origin, implying congenital TB to be merely an etiology rather than a separate clinical entity [7]. Differentiating between congenital and perinatal TB highlights mostly epidemiological differences as presentation, evolution, diagnosis, treatment, and prognosis are analogous [7-8]. Perinatal TB is an equally uncommon infection with approximately 300 reported cases, if examined as a separate entity from congenital TB [9]. Our case illustrates an exceedingly rare presentation of perinatal TB -- a bibliographic search of the PubMed, CNKI, and WANFANG databases revealed no other reported cases of perinatal TB with hepatic, splenic, and peritoneal involvement. Therefore, due to the similarities between congenital and perinatal TB, and scarcity of detailed evidence, we have cited some data from congenital TB studies.

The lungs and liver are the most commonly involved organs in perinatal TB, but the overall clinical picture is often nonspecific. Furthermore, 60%-70% of the mothers had no clinical manifestations of TB and were diagnosed after delivery [10]. Yeh et al. reported a 2.2-fold increase in infant mortality in cases where the mothers had subclinical TB [11]. In the most recent analysis of 92 cases of congenital TB, the reported incidence of splenic TB at 12% and to our knowledge, there are only two cases on PubMed cases reports describing tuberculous peritonitis [10, 12-13]. For our patient, the onset of symptoms at age 25 days was consistent with other reported cases, where the average age on onset of symptoms ranged between 24 and 28 days [3, 14]. The T-cell dependent cellular immune response is diminished during pregnancy which increases both, the likelihood to acquire the infection and reactivation risk of a latent infection due to decreased tumor necrosis factor levels that are required to sustain granulomas [15]. 

A triad of culture negative sepsis, abnormal chest X-ray (diffuse miliary nodules or pneumonia-like changes), and no improvement after standard empiric treatment should immediately raise suspicion for congenital TB. Diagnostic workup should begin with obtaining sputum samples, most commonly via gastric aspiration for the detection of: TB DNA via polymerase chain reaction (PCR), TB culturing, and TB acid-fast staining. PCR detection yields the highest likelihood to be consistently positive (67%), while positive results for cultures and acid fast-staining range between 24%-40% and 10%-50%, respectively, across various studies [16-18]. There is still no definitive information regarding the sensitivity and specificity of classic TST or IFN-γ release assay (IGRA) testing of LTBI in pregnancy -- although both are safe and recommended to screen for LTBI [15]. WHO also recommends to conduct TST and IGRAs (both tests to increase sensitivity) in all children with suspected TB, although evidence for use of both tests in neonates for diagnosing LTBI is limited [5]. A novel strategy to diagnose pulmonary TB in children was recently reported by Zar et al., where the use of the Xpert MTB/RIF Ultra automated nucleic acid amplification assay on combinations of induced sputum and nasopharyngeal aspirate samples obtained a sensitivity and specificity of 80% and 87.5% respectively [19]. When the Xpert MTB/RIF assay was used to diagnose extrapulmonary TB in children, Seo et al. reported a sensitivity and specificity of 80% and 95% for lymph node tissues and aspirates [20].

## Conclusions

Much like systemic lupus erythematosus and syphilis have gained notoriety as the great imitators, perinatal TB should also be approached with the same mindset. Culture negative sepsis in combination with diffuse bilateral pulmonary nodules in an infant should immediately place TB on the differential, despite extensive gastrointestinal involvement or other atypical perinatal TB manifestations. To date, the highest sensitivity and specificity for detecting pulmonary TB in the pediatric population has been achieved with the Xpert MTB/RIF assay on a combination of aspirate samples. If unavailable, PCR of aspirate samples should be the test of choice, as acid-fast smears and culturing have low sensitivity and specificity. If initiated early, treatment with isoniazid, rifampin, pyrazinamide, and either streptomycin or ethambutol has low mortality rates, although the emergence of multidrug-resistant TB strains is a concern. Lastly, the prenatal period should be approached as a unique opportunity to establish an early diagnosis through maternal screening in endemic areas.
